# Synovial Fluid as a Window into Early Cartilage Remodeling After Autologous Matrix-Induced Chondrogenesis

**DOI:** 10.3390/medicina62050922

**Published:** 2026-05-09

**Authors:** Adrian Urbanek, Maciej Wrotniak, Paweł Dolibog, Zenon Czuba, Grzegorz Pilecki, Marcin Kostuj, Paulina Zalejska-Fiolka, Łukasz Polczak, Aleksandra Roubo-Urbanek, Marcin Hajzyk, Jolanta Zalejska-Fiolka

**Affiliations:** 1 Department of Biochemistry, Faculty of Medical Sciences in Zabrze, Medical University of Silesia, 40-055 Katowice, Poland; s91883@365.sum.edu.pl (P.Z.-F.); jzalejskafiolka@sum.edu.pl (J.Z.-F.); 2 Department of Medical Rescue, Faculty of Medical Sciences in Zabrze, Medical University of Silesia, 40-055 Katowice, Poland; 3 Department of Biophysics, Faculty of Medical Sciences in Zabrze, Medical University of Silesia, 40-055 Katowice, Poland; 4 Department of Microbiology and Immunology, Faculty of Medical Sciences in Zabrze, Medical University of Silesia, 40-055 Katowice, Poland; zczuba@sum.edu.pl; 5 Department of Pediatric Orthopedics and Traumatology, Municipal Hospital Complex, 41-500 Chorzów, Polandmarcin.hajzyk@wp.pl (M.H.); 6 Department of Medical and Molecular Biology, Faculty of Medical Sciences in Zabrze, Medical University of Silesia, 40-055 Katowice, Poland; 7 Independent Public Healthcare Unit, Department of Trauma and Orthopedic Surgery, Provincial Specialist Hospital No. 3 in Rybnik, 44-200 Rybnik, Poland; 8 Department of Surgery for Child Developmental Defects and Traumatology, Faculty of Medical Sciences in Zabrze, Medical University of Silesia, 40-055 Katowice, Poland; 9 Scientific Research Facility, Branch in Bielsko-Biała, Medical University of Silesia, 40-055 Katowice, Poland

**Keywords:** AMIC, autologous matrix-induced chondrogenesis, knee, chondral defect, osteochondral defect, synovial fluid, serum, biomarkers, MMP-3, TIMP-2, COMP, PIICP, cartilage remodeling

## Abstract

*Background and Objectives*: Autologous matrix-induced chondrogenesis (AMIC) is an established technique for the treatment of focal cartilage defects of the knee, with well-documented clinical outcomes. However, the biological processes underlying early postoperative cartilage remodeling remain poorly characterized, and the role of synovial fluid biomarkers in this setting is not well defined. This study aimed to assess short-term changes in selected synovial fluid and serum biomarkers of cartilage turnover after AMIC and to examine their associations with clinical outcomes. *Materials and Methods*: Fifteen patients undergoing AMIC for focal knee chondral or osteochondral defects were prospectively enrolled. Synovial fluid and serum samples were collected intraoperatively and at 6 and 12 weeks postoperatively. Concentrations of matrix metalloproteinase-3 (MMP-3), tissue inhibitor of metalloproteinases-2 (TIMP-2), cartilage oligomeric matrix protein (COMP), and procollagen type II C-terminal propeptide (PIICP) were measured using multiplex flow luminescence immunoassay. Clinical outcomes were evaluated using the International Knee Documentation Committee (IKDC) and Lysholm scores preoperatively and at 6 and 12 months. *Results*: Both IKDC and Lysholm scores improved significantly during follow-up. Absolute biomarker concentrations in synovial fluid were low and did not change significantly over time. Nevertheless, higher MMP-3 levels, higher COMP concentrations, and a higher MMP-3/TIMP-2 ratio were associated with poorer clinical improvement. Correlations between synovial fluid and serum biomarker levels were generally weak. Total synovial fluid protein increased postoperatively but did not account for the low biomarker concentrations. *Conclusions*: Early biomarker profiles after AMIC were characterized by low absolute concentrations without significant temporal changes. However, associations of COMP, MMP-3, and the MMP-3/TIMP-2 ratio with clinical outcomes suggest that relative biomarker patterns may reflect early intra-articular remodeling. Synovial fluid analysis may provide more informative insight into local joint biology than serum measurements in this setting. These findings should be interpreted cautiously and require confirmation in larger cohorts.

## 1. Introduction

Articular cartilage injuries of the knee are a common condition identified in approximately 60% of arthroscopies, and often cause persistent pain, swelling, and functional limitation of the joint [1,2]. Untreated defects, apart from being symptomatic, may progress over time and contribute to early degenerative change [3,4,5]. Post-traumatic osteoarthritis (PTOA) is no longer regarded as a purely passive consequence of mechanical wear. Articular surface injury initiates an active mechanobiological response within cartilage and adjacent joint tissues, leading to disturbed extracellular matrix homeostasis, increased metalloproteinase activity, altered balance between MMPs and TIMPs, release of matrix-derived proteins, and abnormal collagen type II turnover, all of which may promote progressive joint degeneration [6,7]. New surgical strategies for cartilage repair continue to be developed, yet no single universally optimal treatment has been established to date.

One of the oldest and still widely used surgical options is microfracture. This technique is based on marrow stimulation and aims to induce migration of progenitor cells from the subchondral bone into the defect. However, the resulting repair tissue is often fibrocartilaginous or mixed in composition and differs from native hyaline cartilage, with a relatively higher proportion of type I collagen and inferior structural organization [8,9]. Autologous matrix-induced chondrogenesis (AMIC) was introduced as an “enhanced” marrow-stimulation strategy combining microfracture with coverage of the defect by a collagen I/III membrane, typically fixed with fibrin glue or sutures, in order to stabilize the marrow clot and support cell retention and matrix deposition [10]. Clinical studies have reported meaningful functional improvement after AMIC, with some evidence of superior mid-term outcomes compared with microfracture alone [11,12].

Second-look data after AMIC remain limited. In a biopsy-based study the overall scar tissue quality was satisfactory, but still usually fibrocartilaginous, rather than uniformly normal hyaline cartilage. The study also suggests that biological maturation after AMIC may continue for more than 12 months [13]. A collagen–hydroxyapatite scaffold was used in the present study because it provides structural support for the marrow clot and a three-dimensional local environment intended to facilitate early repair tissue organization in localized chondral and osteochondral defects. This may also help determine whether synovial fluid biomarkers reflect early intra-articular remodeling after scaffold-assisted repair.

Synovial fluid biomarker analysis offers a complementary window into postoperative joint biology. A recent systematic review showed that synovial fluid biomarkers have been investigated as potential predictors of clinical outcomes after cartilage repair and regeneration procedures [14]. In arthroscopy cohorts, biomarkers directly related to cartilage turnover—particularly MMP-3, TIMP-2, and their relative balance—have been associated with articular pathology and with later patient-reported outcomes, supporting the concept that measurable biochemical activity in synovial fluid may reflect clinically relevant intra-articular remodeling [15,16].

Articular hyaline cartilage is composed predominantly of extracellular matrix rich in proteoglycans and type II collagen, which together determine its load-bearing properties. During cartilage formation, type II procollagen molecules undergo extracellular processing, during which terminal propeptides are cleaved and released into the surrounding biological fluids. The C-terminal propeptide of type II procollagen (PIICP, also referred to as CPII in parts of the literature) therefore reflects type II collagen synthesis and may serve as a marker of anabolic cartilage activity [17,18]. In the context of marrow-stimulation–based repair, a lack of early increase in PIICP may suggest delayed or limited formation of hyaline-like cartilage, whereas elevated formation signals could be consistent with robust anabolic activity.

Cartilage oligomeric matrix protein (COMP) is a non-collagenous extracellular matrix protein abundantly present in cartilage and other joint tissues. Increased COMP concentrations in synovial fluid or serum have been associated with cartilage matrix turnover and joint tissue damage, making COMP a plausible marker of cartilage wear and remodeling burden in focal cartilage defects [19,20].

Matrix metalloproteinase-3 (MMP-3, stromelysin-1) is a key protease involved in degradation of cartilage extracellular matrix. Moreover, it initiates proteolytic cascade by activating other enzymes, including MMP-13, a major collagenase involved in type II collagen degradation [21,22]. Tissue inhibitor of metalloproteinases-2 (TIMP-2) is part of the endogenous counter-regulatory system that restrains metalloproteinase activity and contributes to matrix protection [23]. Because cartilage remodeling is a dynamic process regulated not by isolated concentrations of degradative enzymes or their inhibitors, but by the balance between them, ratios such as MMP-3/TIMP-2 may provide a more integrated estimate of intra-articular catabolic activity than either marker alone. In arthroscopy cohorts, such protease/inhibitor balance has been associated with postoperative outcomes [15].

In this study, we aimed to characterize short-term postoperative profiles of TIMP-2, COMP, MMP-3, and PIICP in synovial fluid and serum in patients undergoing AMIC procedure using a collagen–hydroxyapatite scaffold (MaioRegen ^®^, Finceramica S.p.A., Faenza, Italy) for focal knee chondral and osteochondral defects, and to explore associations between early biomarker signals, and patient-reported outcomes over 12 months. We also sought to compare synovial and systemic measurements to assess whether serum sampling reflects local joint biology in this setting.

This study represents a secondary biomarker analysis of the same prospective AMIC cohort previously examined for inflammatory mediators [24], but addresses a different biological question focused on cartilage remodeling markers and their relationship with clinical outcomes.

## 2. Materials and Methods

### 2.1. Subjects

This prospective observational study was conducted in a cohort of 15 patients undergoing AMIC for focal symptomatic cartilage defects of the knee. The age of the participants ranged from 29 to 57 years, with a median of 44 years and a mean value of 44.1 ± 9.1 years. Body mass index (BMI) values ranged from 21.6 to 35.0 kg/m^2^; the median BMI was 30.0 kg/m^2^, while the mean BMI was 29.1 ± 3.4 kg/m^2^. The size of the chondral defect varied between 0.7 and 4.4 cm^2^, with a median of 2.3 cm^2^ and a mean of 2.3 ± 0.9 cm^2^. These data are presented in Table 1. Lesions were classified as ICRS grade III–IV and included predominantly medial femoral condyle defects. Patients were eligible if they presented with focal, symptomatic cartilage defects of the knee and were scheduled for AMIC with the ability to comply with the follow-up protocol. Exclusion criteria included radiographic or clinical osteoarthritis, age above 60 years, malalignment of the mechanical axis, ligamentous instability, clinically significant meniscal tears, inflammatory or rheumatologic conditions, intra-articular injections within 3 months prior to surgery, previous knee surgery, or any surgical intervention during the follow-up period. All participants provided written informed consent for the use of biological material collected during the procedure. The study protocol was approved by the local Ethics Committee of the Medical University of Silesia (approval code: PCN/CBN/0052/KB1/8/II/22; approval date: 12 July 2022).

### 2.2. Surgical Technique

All patients underwent standard AMIC performed after preoperative clinical and MRI-based assessment of the lesion, followed by intraoperative arthroscopic confirmation of the defect. Following mini-arthrotomy, the lesion was prepared, the sclerotic bed was debrided, and micro-drilling was performed before implantation of the collagen membrane fixed with fibrin glue. In selected cases, concomitant partial meniscectomy or autologous bone grafting was additionally performed. Rehabilitation followed a structured postoperative protocol consisting of protected weight-bearing during the first 6 weeks, progressive range-of-motion and strengthening exercises, and return to sports only after functional recovery and clinical approval.

### 2.3. Outcomes of Interest

Clinical outcomes were assessed using IKDC and Lysholm scores obtained preoperatively and at 6 and 12 months. For the present analysis, the primary biochemical objective was to characterize short-term profiles of synovial fluid and serum cartilage remodeling markers and to relate these measurements to later clinical improvement.

Sampling time points were identical to those used in the related inflammatory biomarker study [24]: intraoperatively before AMIC, and at 6 and 12 weeks postoperatively. Synovial fluid was collected without lavage, centrifuged immediately, and stored at −83 °C until analysis. Serum was collected at the same time points. Concentrations of MMP-3, TIMP-2, COMP, and PIICP were measured in a single analytical run using a multiplex Flow Luminescence Immunoassay (FLIA) according to the manufacturer’s protocol (Cloud-Clone Corp., Katy, TX, USA). All samples were collected, processed, and analyzed under identical conditions, ensuring that any systematic underestimation affected all measurements equally and allowing valid relative comparisons across subjects and time points. Total protein concentration in synovial fluid was determined using the biuret method in accordance with standard laboratory procedures.

Clinical outcome measures which have been previously reported for this cohort, are included in the present study for contextual purposes only and therefore are presented in the Appendix A.

### 2.4. Statistical Analysis

Statistical analyses were performed using Statistica 13.3 (TIBCO Software Inc., Palo Alto, CA, USA, 2017) and the R environment with RStudio (version 2026.01.1 Build 403). Quantitative variables were presented as mean ± standard deviation (SD) and minimum and maximum values (min–max). The distribution of the analyzed variables was assessed using the Shapiro–Wilk test, which demonstrated that the assumption of normality was not met for the examined variables. Therefore, non-parametric statistical methods were applied. Changes in clinical scores (IKDC, Lysholm) were evaluated across baseline, 6 months, and 12 months, whereas biochemical markers (COMP, PIICP, MMP-3, TIMP-2, total protein) were analyzed across baseline, 6 weeks, and 12 weeks using the Friedman test. When a significant main effect of time was detected, post-hoc analysis was performed using the Dunn test with Bonferroni–Holm correction to control the type I error rate associated with multiple comparisons. Effect size for the Friedman test was assessed using Kendall’s coefficient of concordance (W), which ranges from 0 to 1 and reflects the degree of rank agreement between repeated measurements. Values of W ≈ 0.1 were interpreted as indicating a small effect, W ≈ 0.3 as a moderate effect, and W ≥ 0.5 as a large effect. Associations between clinical scales and biochemical markers were assessed using Spearman’s rank correlation coefficients. Correlation results were reported as rho values with corresponding *p*-values and presented graphically as a heat map to enable visual assessment of the strength and direction of relationships between variables. A significance level of *p* < 0.05 was considered statistically significant.

## 3. Results

### 3.1. Clinical Outcomes

Clinical outcomes demonstrated significant improvements in both Lysholm and IKDC scores across the postoperative follow-up. For the Lysholm knee score, the median value increased from 62.0 preoperatively to 72.0 at 6 months and 83.0 at 12 months (*p* = 0.00033). The mean Lysholm score also improved from 57.5 ± 18.6 before surgery to 70.3 ± 15.9 at 6 months and 78.3 ± 14.7 at 12 months. Post-hoc comparisons using the Dunn–Bonferroni-Holm test showed significant improvements from baseline to 6 months (*p* = 0.01593) and from baseline to 12 months (*p* = 0.00048). No significant difference was observed between 6 and 12 months (*p* = 0.26157). Similarly, the IKDC score showed a marked growth over time, with the median increasing from 28.0 preoperatively to 55.0 at 6 months and 64.0 at 12 months (*p* = 0.00037). Corresponding mean values improved from 30.6 ± 9.4 to 51.8 ± 15.2 and 58.8 ± 15.0, respectively. Post-hoc comparisons showed statistically significant improvements between baseline and 6-month scores (*p* = 0.00227) and between baseline and 12-month scores (*p* = 0.00227). Differences between 6 and 12 months (*p* = 1) did not reach statistical significance. Overall, both PROMs demonstrated statistically and clinically meaningful improvements following surgery. Detailed descriptive statistics are provided in Appendix A Table A1.

### 3.2. Biomarker Levels in Synovial Fluid

Synovial fluid concentrations of COMP and TIMP-2 (ng/mL), MMP-3 and PIICP (pg/mL), and total protein (g/L) were assessed at baseline (intraoperatively prior to cartilage repair), and at 6 and 12 weeks postoperatively.

**Figure 1:**
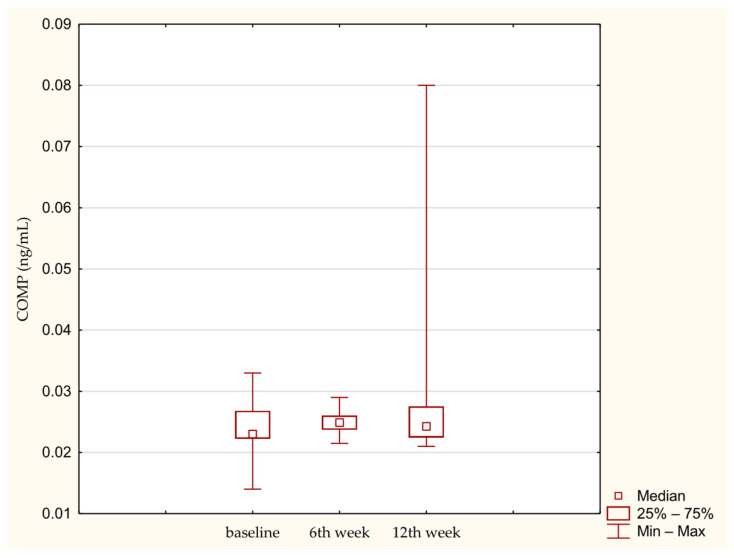
COMP concentration in synovial fluid at baseline. 6th week. 12th week. Abbreviations: 25%—first quartile; 75%—third quartile; min—minimum value; max—maximum value.

**Figure 2:**
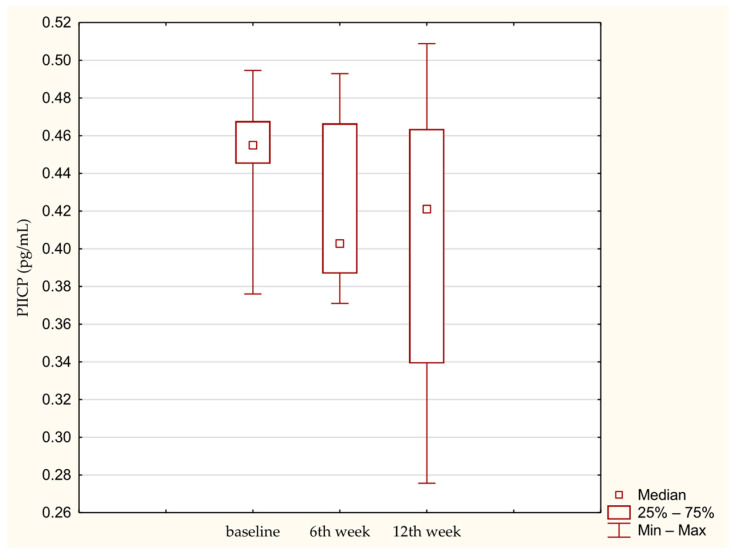
PIICP concentration in synovial fluid at baseline. 6th week. 12th week. Abbreviations: 25%—first quartile; 75%—third quartile; min—minimum value; max—maximum value.

Overall absolute concentrations of remodeling biomarkers were low across all time points. No significant time-dependent changes were observed for COMP (Figure 1) or PIICP (Figure 2).

**Figure 3:**
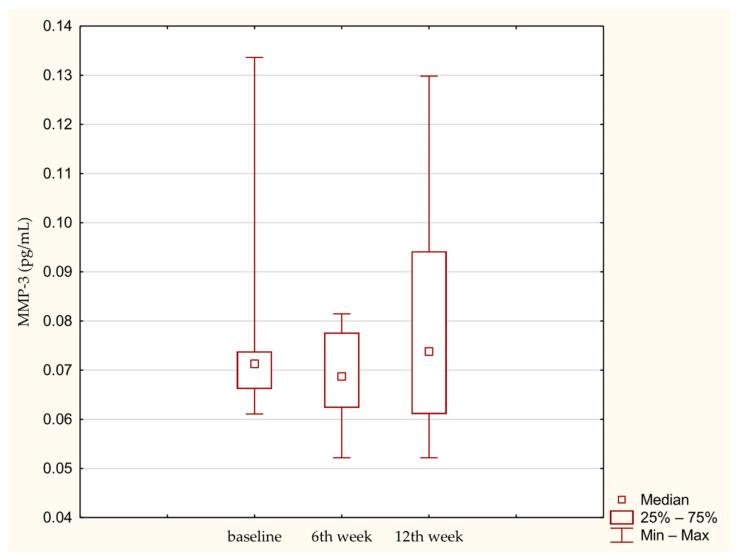
MMP-3 concentration in synovial fluid at baseline. 6th week. 12th week. Abbreviations: 25%—first quartile; 75%—third quartile; min—minimum value; max—maximum value.

**Figure 4:**
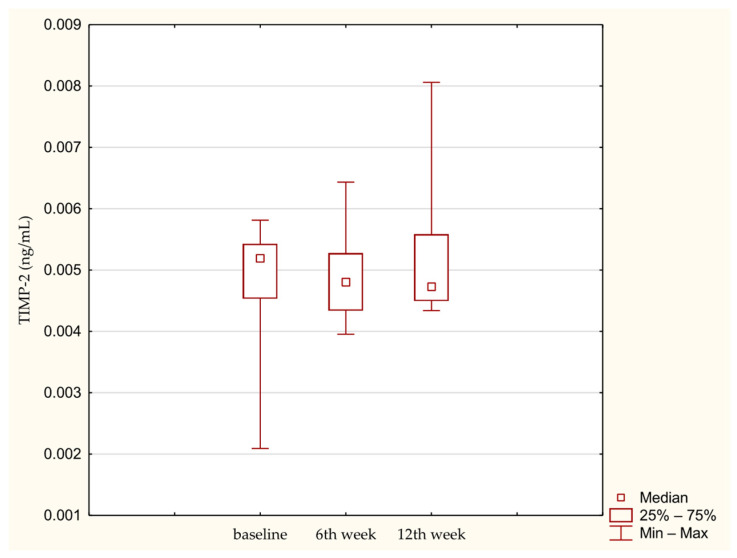
TIMP-2 concentration in synovial fluid at baseline. 6th week. 12th week. Abbreviations: 25%—first quartile; 75%—third quartile; min—minimum value; max—maximum value.

Similarly MMP-3 did not change significantly over time (Figure 3). TIMP-2 also showed no significant increase during the 12-week observation period (Figure 4). Effect size estimation using Kendall’s coefficient of concordance indicated a small effect for PIICP, and very small effects for COMP, MMP-3 and TIMP-2, consistent with limited temporal variability of these biomarkers during the early postoperative period.

Total protein concentration in synovial fluid was analyzed as a comparator for biomarker levels and as an indicator of sample quality. Protein levels increased over time, as previously described [24], and are presented here for contextual reference only. In contrast to the biomarker measurements, total protein exhibited a large effect size, while remaining within a physiological range and demonstrating a relatively consistent distribution across time points, supporting adequate sample quality. The results are summarized in Table 2. Importantly, these findings do not explain the low absolute concentrations of the analyzed biomarkers, and normalization of biomarker levels to total protein content (biomarker-to-protein ratio) would not be expected to alter the statistical significance of the observed results.

The increase in total protein concentration may reflect mild postoperative synovial response; however, it does not appear to account for the variability or magnitude of biomarker levels observed in this study.

### 3.3. Correlations Between Biomarkers in Synovial Fluid and Serum

Correlations between serum and synovial fluid biomarker levels at corresponding time points were generally weak. The only significant correlation between compartments for an individual biomarker was observed for PIICP (ρ = −0.7568). This finding aligns with results reported by Garvican et al. in which serum PIICP levels in patients with osteoarthritis were found to be decreased compared with those in healthy individuals [25]. Despite this, serum biomarker levels generally did not reflect the local intra-articular environment and were therefore not further analyzed in relation to clinical outcomes. Data on serum biomarker concentrations are presented in Appendix A.1.

### 3.4. Correlations Between Preoperative Clinical Status and Synovial Fluid Biomarkers

**Figure 5:**
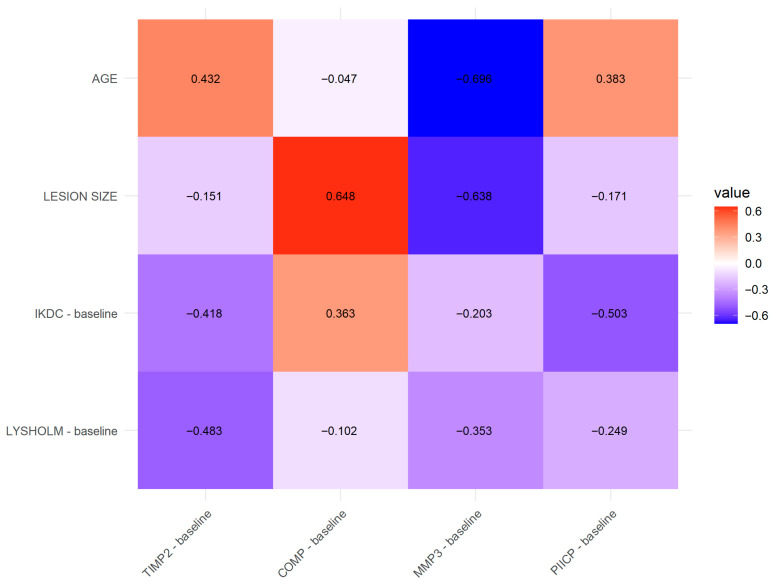
Heatmap illustrating Spearman’s rank correlations (ρ) between biomarker concentrations and preoperative demographic data and clinical status. Color intensity reflects the strength of the correlation (ρ), with warmer colors indicating positive correlations and cooler colors indicating negative correlations.

Preoperative demographic parameters and patient-reported outcome measures (PROMs) were correlated with baseline synovial fluid biomarker levels. The only significant association was observed between cartilage defect size and COMP concentration (ρ = 0.6480, *p* < 0.05). As previously described, COMP is released during cartilage matrix degradation and may serve as a marker of cartilage breakdown. This finding supports the concept that greater structural damage is associated with increased COMP levels in synovial fluid. The magnitude and direction of these associations are illustrated in the correlation heatmap (Figure 5).

### 3.5. Correlations Between Biomarkers and Clinical Outcomes

To evaluate the relationship between the early postoperative joint environment and surgical outcome, changes in PROMs between baseline and 12 months were correlated with synovial fluid biomarker levels measured at 6 and 12 weeks after the procedure. Given the observational design, limited sample size and multiple comparisons, these associations should be interpreted cautiously. In addition, synovial fluid concentrations of TIMP-2, COMP, MMP-3 and PIICP were relatively low, with most calibrated values falling below the assay’s lower limit of detection. Despite these low absolute concentrations, consistent and statistically significant associations with clinical outcomes (IKDC and Lysholm scores), cartilage defect size, and the MMP-3/TIMP-2 ratio were observed, suggesting that these values may carry meaningful relative biological information reflecting early intra-articular remodeling following AMIC.

A significant negative correlation was found between both the MMP-3/TIMP-2 ratio (ρ = −0.636, *p* < 0.05) and MMP-3 levels (ρ = −0.678, *p* < 0.05) and improvement in IKDC score, which may indicate the presence of more pronounced early catabolic activity in patients with poorer clinical outcomes.

Similarly higher COMP levels were negatively correlated with postoperative improvement in both IKDC and Lysholm scores (ρ = −0.596 and ρ = −0.604 respectively; *p* < 0.05). This may reflect greater baseline cartilage damage, increased mechanical stress within the joint, or more intensive early extracellular matrix turnover. and is consistent with the observed catabolic activity represented by the MMP-3/TIMP-2 ratio.

No significant correlations were observed for PIICP at any analyzed time point. In combination with the lack of a significant increase in PIICP over time. this may suggest either limited early formation of hyaline-like cartilage following AMIC or a limited sensitivity of this biomarker for detecting early cartilage regeneration.

**Figure 6:**
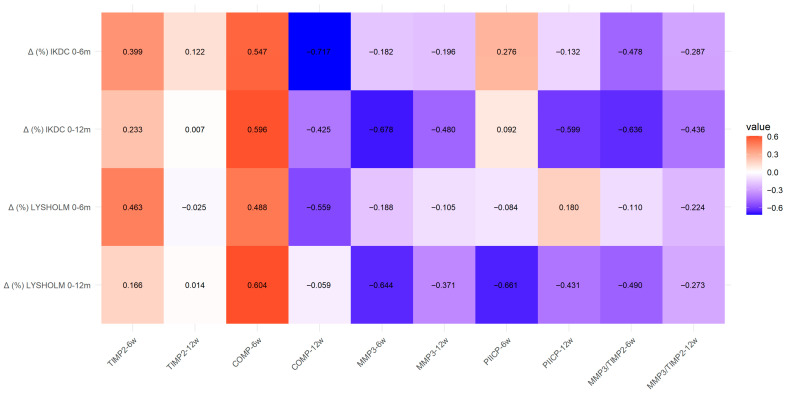
Heatmap illustrating Spearman’s rank correlations (ρ) between biomarker concentrations and MMP-3/TIMP-2 ratio and changes in clinical scores (Δ). Color intensity reflects the strength of the correlation (ρ), with warmer colors indicating positive correlations and cooler colors indicating negative correlations.

The biomarker–outcome associations are illustrated in the heatmap below (Figure 6).

## 4. Discussion

Published AMIC literature has focused predominantly on clinical outcomes, imaging assessment, and, in selected reports, second-look or histological findings whereas serial synovial fluid profiling of cartilage remodeling biomarkers after AMIC has remained largely unexplored. In the present cohort, both IKDC and Lysholm scores improved to an extent exceeding established minimal clinically important difference thresholds, indicating that the procedure provided clinically meaningful short-term benefit [26]. These favorable functional results are consistent with previously published cartilage repair studies, but they do not explain which intra-articular biological processes accompany early postoperative recovery. For this reason synovial fluid analysis was included as a complementary approach to clinical assessment.

The biomarker panel used in this study was selected to reflect complementary aspects of cartilage remodeling. COMP was included as a marker of cartilage matrix disruption and joint tissue wear since increased release of COMP into synovial fluid has been described after knee injury and in early osteoarthritic change [19,27]. PIICP was chosen as an indicator of type II collagen synthesis and therefore as a marker of anabolic activity, which is particularly relevant in AMIC, where the quality of the newly formed tissue remains a key biological question [17,25]. MMP-3 was included because it plays a central role in cartilage degeneration, taking part in initiating proteolytic cascade within the joint. Although MMP-13 is more specific to type II collagen degradation, MMP-3 was considered more informative in this setting, as it reflects broader matrix remodeling activity and can be interpreted in conjunction with TIMP-2 to assess the overall balance between degradative and inhibitory processes [28,29,30].

Direct comparisons with previous studies are challenging, because synovial fluid biomarker research in cartilage repair remains limited and methodologically heterogeneous, with substantial variability in procedure type, sampling intervals and analytical methods [14]. In a mixed knee arthroscopy cohort Cuéllar et al. demonstrated that synovial fluid biomarkers including MMP-3 and TIMP-2 were associated with clinical status and postoperative outcomes, supporting the concept that these markers reflect the intra-articular biological environment [15]. In contrast, Anil et al. reported an early postoperative increase in MMP-3 accompanied by a decrease in TIMP-2 within approximately 10 days after anterior cruciate ligament reconstruction, suggesting that protease-dominant activity may be more pronounced in the immediate postoperative phase [31]. This difference in timing is particularly relevant as studies sampling synovial fluid within days of surgery are likely to capture transient inflammatory and catabolic peaks that may no longer be detectable several weeks later.

Evidence from cartilage repair procedures remains inconsistent. In patients undergoing autologous chondrocyte implantation Schneider et al. observed an initial postoperative increase in cartilage degradation markers followed by a decline below baseline levels at 3 to 6 months, indicating dynamic but time-dependent remodeling activity [32]. Vasara et al. reported that synovial fluid MMP-3 levels were higher in patients with cartilage lesions than in control knees, and remained elevated one year after autologous chondrocyte transplantation, which was interpreted as reflecting ongoing graft remodeling or early degeneration [33]. In contrast to that observation, we did not identify a clear relationship between MMP-3 and baseline lesion characteristics in our cohort. In a cohort undergoing microfracture or osteotomy MMP-3 or COMP were not identified as significant predictors of clinical outcome, suggesting that the prognostic value of individual biomarkers may be limited and dependent on procedure performed or composite biomarker profiles [34]. Similarly Schmidt-Rohlfing et al. reported no significant correlation between MMP-3 or COMP and cartilage lesion severity assessed arthroscopically, further indicating that these markers may not reliably reflect structural cartilage damage when considered in isolation [35].

With regard to the relationship between synovial fluid and serum biomarkers Catterall et al. demonstrated that synovial fluid concentrations of MMP-3 exceeded those observed in serum and showed a significant correlation between compartments for MMP-3, whereas no such relationship was observed for COMP, supporting the concept that local intra-articular processes are only partially reflected in systemic measurements [36]. Bobacz et al. reported a significant correlation between serum and synovial fluid proMMP-3 levels in patients with knee injury, although this relationship was not associated with the severity of cartilage damage indicating that systemic measurements may capture only selected aspects of local joint metabolism [37]. Taken together, the available literature indicates that synovial fluid biomarkers of cartilage remodeling are strongly dependent on timing biological context and methodological factors. Therefore the absence of significant longitudinal changes in the present study should not be interpreted as a lack of biological activity. Rather the observed associations between higher MMP-3 higher COMP and an unfavorable MMP-3/TIMP-2 balance with poorer clinical outcomes suggest that even low absolute biomarker levels may still reflect biologically relevant differences in early intra-articular remodeling processes.

An important consideration in the interpretation of the present results is that a substantial proportion of measured biomarker concentrations were close to or below the lower limit of detection of the assay. This issue is not uncommon in synovial fluid biomarker studies particularly when multiplex platforms are used and analyte concentrations are low. In these situations absolute values should be interpreted with caution; however this does not rule out the possibility of identifying meaningful relative differences within a cohort analyzed under the same conditions. In the present study all samples were collected, handled and analyzed under identical conditions and the observed patterns, including correlations with clinical outcomes and internal consistency between time points suggest that the measurements may still reflect underlying intra-articular processes, despite their low absolute magnitude. Therefore rather than focusing on absolute concentrations alone, the results should be interpreted in the context of relative differences and biomarker relationships, which may be more informative in early postoperative settings.

## 5. Limitations

This study has several limitations. First, no control cohort was included. Direct comparison with healthy synovial fluid is difficult because aspiration from unaffected joints is technically challenging, often yields very small sample volumes, and repeated sampling at three time points in asymptomatic individuals raises ethical concerns.

The relatively small cohort size (*n* = 15) restricts statistical power and may limit the applicability of the findings particularly in the context of correlation analyses. Interpretation of the findings is also limited by between-subject heterogeneity, including variation in age, BMI, and defect size, which may have influenced biomarker distributions and clinical response patterns. In addition, some patients underwent concomitant procedures. Limited partial meniscectomy was performed in seven patients and autologous bone graft augmentation in one patient, which may have introduced variability in the intra-articular environment and influenced biomarker expression. Because of the small sample size, subgroup or sensitivity analyses according to baseline characteristics or concomitant procedures were not feasible.

An additional limitation relates to the low absolute concentrations of the analyzed biomarkers, with many values approaching or falling below the lower limit of detection of the assay. This limits the interpretation of absolute biomarker levels and may affect the precision of quantitative assessment. Although all samples were processed under standardized conditions, these findings should be interpreted with caution; however, the analyses were performed by experienced laboratory personnel, reducing the risk of analytical variability.

## 6. Conclusions

In this exploratory study, synovial fluid biomarker profiling after AMIC procedure revealed low absolute concentrations of TIMP-2, COMP, MMP-3 and PIICP with no significant time-dependent changes during the early postoperative period. Despite this, consistent associations between selected biomarkers—particularly MMP-3, COMP and the MMP-3/TIMP-2 ratio—and clinical outcomes were observed, suggesting that these parameters may reflect aspects of early intra-articular remodeling. In addition, larger cartilage defects were associated with higher baseline synovial fluid COMP concentrations, supporting the relationship between structural damage and local matrix turnover.

The findings indicate that synovial fluid biomarkers, even at low concentrations may provide additional information beyond clinical assessment alone and could contribute to the understanding of early biological processes following cartilage repair.

Further studies in larger, more homogeneous cohorts with extended follow-up and standardized biomarker assessment are required to confirm these observations and to better define the clinical utility of synovial fluid biomarkers in cartilage repair procedures.

## Figures and Tables

**Table 1:** Demographic data and characteristics of cartilage defects in the study cohort.

Variable	Mean	SD	Median	MIN	MAX
Age (years)	44.1	9.1	44.0	29.0	57.0
BMI (kg/m^2^)	29.1	3.4	30.0	21.6	35.0
Defect size (cm^2^)	2.3	0.9	2.3	0.7	4.4

**Table 2:** Biomarker concentrations in synovial fluid.

Concentration of Biomarker	Timepoint	Mean	SD	Median	Min	Max	*p*
COMP (ng/mL)	Pre-operative	0.0236	0.0050	0.0230	0.0140	0.0330	0.45943
	6th week post-op	0.0250	0.0022	0.0250	0.0215	0.0290	
	12th week post-op	0.0291	0.0162	0.0243	0.0210	0.0800	
PIICP (pg/mL)	Pre-operative	0.4502	0.0319	0.4550	0.3760	0.4946	0.19691
	6th week post-op	0.4252	0.0466	0.4028	0.3710	0.4929	
	12th week post-op	0.4040	0.0869	0.4211	0.2756	0.5088	
MMP-3 (pg/mL)	Pre-operative	0.0795	0.0269	0.0713	0.0611	0.1336	0.62270
	6th week post-op	0.0690	0.0096	0.0687	0.0522	0.0815	
	12th week post-op	0.0802	0.0265	0.0738	0.0522	0.1298	
TIMP-2 (ng/mL)	Pre-operative	0.0048	0.0011	0.0052	0.0021	0.0058	0.73380
	6th week post-op	0.0049	0.0008	0.0048	0.0040	0.0064	
	12th week post-op	0.0052	0.0010	0.0047	0.0043	0.0081	
Protein total (g/L)	Pre-operative	31.7060	8.2275	32.9883	20.6852	49.0926	0.00043
	6th week post-op	44.8341	8.7489	43.7376	29.4526	60.8477	
	12th week post-op	42.7972	8.5670	42.6206	23.9254	53.6568	

## Data Availability

The data presented in this study are available on reasonable request from the corresponding author. The data are not publicly available due to privacy and ethical restrictions related to patient confidentiality and institutional review board regulations.

## Abbreviations

The following abbreviations are used in this manuscript:

AMIC	Autologous matrix-induced chondrogenesis
OA	Osteoarthritis
SF	Synovial fluid
PTOA	Post-traumatic osteoarthritis
ACI	Autologous chondrocyte implantation
IKDC	International Knee Documentation Committee
ECM	Extracellular matrix
ICRS	International Cartilage Repair Society
BMI	Body mass index
PROMs	Patient-reported outcome measures
HTO	High tibial osteotomy
MCID	Minimal clinically important difference
FLIA	Flow luminescence immunoassay
LLD	Lower limit of detection
MMP-3	Matrix metalloproteinase-3
TIMP-2	Tissue inhibitor of metalloproteinases-2
COMP	Cartilage oligomeric matrix protein
PIICP	Procollagen type II C-terminal propeptide

## Appendix A

### Appendix A.1

**Table A1:** Lysholm knee score and IKDC score.

SCALE (Point)	Timepoint	Mean	SD	Median	Min	Max	*p*
Lysholm knee score	Pre-operative	57.5	18.6	62.0	23.0	84.0	0.00033
	6th month post-op	70.3	15.9	72.0	41.0	88.0	
	12th month post-op	78.3	14.7	83.0	52.0	100.0	
IKDC score	Pre-operative	30.6	9.4	28.0	17.0	56.0	0.00037
	6th month post-op	51.8	15.2	55.0	27.0	69.0	
	12th month post-op	58.8	15.0	64.0	25.0	74.0	

**Table A2:** Concentrations of MMP-3, TIMP-2, COMP and PIICP in serum.

Concentration of Biomarker	Timepoint	Mean	SD	Median	Min	Max	*p*
COMP (ng/mL)	Pre-operative	0.0225	0.0060	0.0200	0.0180	0.0400	0.03638
	6th week post-op	0.0626	0.0939	0.0240	0.0190	0.3300	
	12th week post-op	0.0213	0.0009	0.0210	0.0200	0.0230	
PIICP (pg/mL)	Pre-operative	1.5953	1.5264	1.0900	0.1802	4.1200	0.41686
	6th week post-op	1.4511	1.2629	1.2723	0.1696	3.0100	
	12th week post-op	1.2884	1.5837	0.4823	0.2120	5.0300	
MMP-3 (pg/mL)	Pre-operative	0.1367	0.2378	0.0490	0.0280	0.8600	0.20276
	6th week post-op	0.4461	1.3214	0.0560	0.0407	4.8400	
	12th week post-op	0.0553	0.0180	0.0484	0.0433	0.1120	
TIMP-2 (ng/mL)	Pre-operative	0.0038	0.0010	0.0036	0.0028	0.0065	0.07001
	6th week post-op	0.0045	0.0018	0.0039	0.0029	0.0090	
	12th week post-op	0.0035	0.0004	0.0036	0.0026	0.0042	
